# The outcome of control groups in clinical trials of conservative treatments for chronic mechanical neck pain: a systematic review

**DOI:** 10.1186/1471-2474-7-58

**Published:** 2006-07-18

**Authors:** Howard Vernon, B Kim Humphreys, Carol Hagino

**Affiliations:** 1 Department of Graduate Education and Research, Canadian Memorial Chiropractic College, Toronto, Ontario, Canada

## Abstract

**Background:**

Chronic neck pain is highly prevalent in Western societies, with about 15% of females and 10% of males suffering with it at any time. The course of untreated chronic neck pain patients in clinical trials has not been well-defined and the placebo effect has not been clarified.

**Methods:**

A systematic review of RCT's of conservative treatments for chronic mechanical neck pain was conducted. Studies were excluded if they did not include a control group, if they involved subjects with whiplash injuries, a predominance of headache or arm pain associated with chronic neck pain and if only one treatment was reported. Only studies scoring 3–5 out of 5 on the Jadad Scale for quality were included in the final analysis. Data on change in pain scores of subjects in both placebo (PL) as well as no-treatment (NT) control groups were analyzed. Mean changes in pain scores as well as effect sizes were calculated, summarized and compared between these groups.

**Results:**

Twenty (20) studies, 5 in the NT group and 15 in the PL group, with outcome intervals ranging from 1–52 weeks were included in the final analysis. The mean [95% CI] effect size of change in pain ratings in the no-treatment control studies at outcome points up to 10 weeks was 0.18 [-0.05, 0.41] and for outcomes from 12–52 weeks it was 0.4 [0.12, 0.68]. In the placebo control groups it was 0.50 [0.10, 0.90] at up to 10 weeks and 0.33. [-1.97, 2.66] at 12–24 weeks. None of the comparisons between the no-treatment and placebo groups were statistically significant.

**Conclusion:**

It appears that the changes in pain scores in subjects with chronic neck pain not due to whiplash who are enrolled in no-treatment and placebo control groups were similarly small and not significantly different. As well, they do not appear to increase over longer-term follow-up.

## Background

Neck pain is a very common problem, second only to low back pain in its frequency in the general population [1-3] and in musculoskeletal practice [4]. Estimates of the prevalence of chronic neck pain vary. In a Swedish population [5] 18.5% of females and 13.2% of males had neck pain for longer than 6 months; however, when continuous chronicity was rated, these figures were reduced to 10% and 7%, respectively. A Finnish study [6] reported chronic neck pain in 13.5% of females and 9.5% of males. A Norwegian study [7] reported an overall rate of 13.8% for neck pain greater than 6 months duration; however, for sub-groups above the age of 43, the rate rose above 20%. It would appear that about 15% of females and 10% of men suffer with chronic neck pain at any given time.

Clinical researchers are interested in the following question as it relates to chronic neck pain: "in a clinical trial, what is the expected outcome of chronic mechanical neck pain patients who are assigned to control groups?" Specifically, it would be useful to know the average magnitudes of change in various clinically important parameters such as pain and disability status at, for example, one, three, six and twelve months, in order to track the course of the condition in the absence of any formal treatment. Surprisingly, this question has received little attention in the literature.

As noted above, numerous studies exist which provide information on the prevalence of chronic neck pain in general and specific (i.e., occupational) populations and typically report these as one-week, one-month, six-month, annual or lifetime occurrences [1-11]. However, the clinical course of these types of patients in the absence of any formal treatment has not been studied so thoroughly. Recently, Borghouts et al. [12] reviewed a selected group of observational studies (with no treatment provided) [13-19] as well as randomized clinical trials (RCT's) which provided information on the clinical course or prognosis of chronic neck pain. They remarked that the body of reports they reviewed had numerous deficiencies. They summarized these studies as follows:

"...within six months, approximately 50% of patients had less pain and a general improvement of 50%, with a mean reduction of pain and use of analgesics of about 30%" [[12], pg.12].

We have used Borghouts et al.'s study as a foundation for an expanded review of the issue of the response of chronic mechanical neck pain patients in clinical trials who do not receive any formal treatment. It is recognized that data obtained from controlled clinical trial reports would not be truly observational, in that subjects in control groups are highly selected for inclusion. As well, they may benefit from the mere fact that they participated in a clinical trial; however, at least this effect would not be confounded by any formal intervention (although, see Discussion, re: self-treatments).

Two types of control groups exist for this purpose: no-treatment (NT) controls and placebo (PL) controls. Each of these groups has advantages and disadvantages with respect to a determination of clinical outcome or course. For no-treatment controls, a so-called "trial effect" (non-specific effect) would exist resulting from the basic fact of selection for and participation in a clinical trial; however, since subjects would be aware of their status as "not receiving treatment", the positive "trial effect" might be counteracted (nocebo effect), making their outcomes an approximation of the "natural history" of chronic neck pain over the duration of the study and any follow-up intervals. In Borghouts' et al. review of 17 RCT's, 3 trials had no-treatment control groups [20-22]; however, the authors do not summarize the outcomes of these studies specifically.

The outcomes of placebo control groups would not constitute the "natural history" of the chronic neck pain, as, in these circumstances, subjects are lead to believe that they are receiving a true treatment [23,24]. Data from placebo groups actually answers the question, "what is the expected outcome over a certain period of time when a person (or a group of similar persons) believes they are receiving true treatment, when, in fact, they are not?" In Borghouts et al., 6 trials are reviewed with placebo controls [25-30], but they, too, were not summarized with respect to this question.

Current experimental theory [23,24] would suggest that the magnitudes of positive change in outcomes of placebo control groups would exceed those of the non-treatment control groups. To our knowledge, however, a systematic analysis of change scores of pain measurements in these control groups of chronic neck pain patients has not specifically been conducted. We report here on a systematic review of conservative intervention studies for chronic mechanical neck pain that have employed either no-treatment or placebo control groups and which provide data which can be applied to the second and third methodologies described above. Thus, we present data from a group of controlled RCT's for chronic neck pain in order to better determine the magnitude of clinical change in chronic mechanical neck pain patients enrolled in clinical trials. It is expected that this will provide as estimate of the placebo effect in these subjects as well as provide useful input for future sample size calculations in this area.

## Methods

A comprehensive literature search was performed in MEDLINE, CINHAHL, AMED, MANTIS, Index to Chiropractic Literature, Alt HealthWatch, the Cochrane Database of Systematic Reviews, the Cochrane Controlled Trials Registry, and several EBSCO Information Services databases (Biomedical Reference Collection, Nursing and Allied Health Collection, Psychological and Behavioural Sciences Collection) using the strategy outlined in Table 1.

Citation searches were also conducted manually. The limitation to English was based on the inability to translate any non-English studies.

Selections were then made according to the criteria described below. *Inclusion*: the study design was a randomized clinical trial of conservative or complementary therapies for chronic neck pain. As several studies employed time frames for pain duration less than the conventional interval of 12 weeks, and as we sought the largest sample possible, we defined "chronic neck pain" as neck pain of longer than eight weeks in duration [31]. Only studies with control groups designated as "no-treatment" (NT) or "placebo" (PL) were included. *Exclusion*: studies involving subjects with a predominance of radicular pain, osteoarthritis of the neck, or headaches were excluded; studies involving subjects with neck and back pain, where the data related to neck pain could not be extracted, were excluded; studies employing exclusively surgery were excluded.

Studies qualifying according to the above criteria were then assessed for quality using the Jadad Scale [32]. The Jadad scale is a 5-point scale which is one of the oldest [32], most well-validated [33,34] and reliable [35] scales for assessing the quality of randomized clinical trials. Studies scoring 3 – 5 out of 5 were accepted.

Data on pain outcomes of the control groups were extracted from each study. Most studies employed a 100 mm visual analogue scale (VAS) to measure pain ratings and reported these as mean (SD) values at baseline and at various outcomes points thereafter. Some trials reported the median values of this outcome. Mean (95%CI) or median values for pain scores were abstracted. Baseline pain scores were averaged and compared between the NT and PL groups with a Student's t-test. Absolute and relative change scores were calculated in mm of a pain visual analogue scale (VAS) and percentages, respectively. These were also compared between the NT and PL groups with a Student's t-test for assumed unequal variances. Effect sizes were calculated when the data were expressed as means (95%CI). Effect size was calculated as the difference between pre- and post-intervention means (for each control group) divided by the pooled standard deviation [36,37].

## Results

The literature search retrieved 1,980 studies, 30 of which met our initial inclusion criteria. After quality ratings, 10 of these were rejected (NT controls = 4 [21,22,39,42], PL controls = 6 [20,27,38,44,48,56]). Twenty studies, 5 in the NT group [40,41,43,57,58] and 15 in the PL group [28-30,46,47,49-55,59-61], were included in our final selection. In this set of studies, the comparison treatments were acupuncture (n = 4), exercise therapy (n = 5), laser therapy (n = 5) electrotherapy (n = 3), magnetic necklace (n = 1), massage (n = 1) and botox injection (n = 1). No study employing strictly medication(s) was found that met our criteria for inclusion with respect to the appropriate clinical characteristics.

Five studies were identified from the search which employed NT control groups [40,41,43,57,58]. In three of these studies, the control condition consisted of no formal intervention at all [41,43,57]. One study used low-level education in the form of informal sessions, once weekly for ten weeks [40]. Finally, one study [58] used minimal infrared therapy and advice. The average age of subjects in this group was 41.2 years. The average baseline pain score was 46.2 mm/100.

The outcome intervals range from 6–52 weeks. Table 2 presents the changes in pain scores of five of the studies with NT controls. Three studies provided data for 1–10 weeks; 2 studies provided data from 12–52 weeks.

The 1–10-week outcomes are summarized as follows: the mean (95%CI) absolute change in VAS mm was -5.7 [-8.6, -2.8] mm; the mean (95%CI) relative change in VAS mm was -10.3% [-14.4, -6.2]; the mean [95%CI] effect size of these changes was 0.18 [-0.05, 0.41].

The 12–52 week outcomes are summarized as follows: the mean absolute change in VAS mm was -11.9 [-17.4, -6.4]; the mean relative change in VAS mm was -25.7% [-34.3, -17.1]; the mean [95% CI] effect size of these changes was 0.4 [0.12, 0.68].

One study provided data on self-rated improvement (% of subjects who rated full improvement). Taimela et al. [41], reported a figure of 23% at 12 weeks and 30% at 52 weeks.

Table 3 presents the results of 14 studies of chronic neck pain patients from the search which employed a PL control group (Lewith and Machin, [46] is not included as it did not report pain scores). All but two of these studies employed placebo versions of acupuncture or electro-physiological therapies such as laser, infra-red and electromagnetic therapies. Hong et al. [60] employed a placebo version of a magnetic necklace while Wheeler et al. [61] employed a single placebo injection into the cervical paraspinal muscles. Rigato et al. [47], divided the subjects into severity categories of mild, moderate and severe, thus producing three separate outcome comparisons for their single placebo group. Lewith and Machin [46] did not provide mean pre-post-intervention outcome scores; rather, they reported on post-treatment self-rated improvement (See Table 3). The average age of the subjects in this group was 43.9. One study [49] involved older patients (average age = 61) who had cervical osteoarthritis. The average baseline pain score in the PL groups was 56.6 mm/100.

The majority of these comparisons were made at between 2–6 weeks post-baseline. Four (4) studies measured outcomes past 10 weeks [28,51,59,61]. At the outcome point up to 10 weeks, in 14 studies, the PL groups showed a mean change of -5.0 mm [-8.1, -1.9] on a 100 mm pain VAS. None showed a mean change larger than 20 mm. The mean relative change was -10.6% [-16.4, -4.8]. The mean [95% CI] effect size in this group of studies was 0.5 [0.10, 0.90].

Four (4) studies [28,51,59,61] provided outcomes from 12–24 weeks. The mean absolute change was -6.7 [-18.6, 5.2] mm and the mean relative change was -8.3% [-32.4, 15.8]. The mean [95%CI] effect size in this group of studies was 0.33 [-1.97, 2.66].

Lewith and Machin [46] reported that 33% of their placebo group rated their level of improvement as "good". This resulted from a change of 2 or more points on a 7-point pain scale (i.e., greater than 29% improvement in self-rated pain). It is not possible to compare this finding with the mean change scores of the other studies. The finding, in Sator-Katzenschlager et al. [50] of an effect size of 5.3 after six weeks was considered an anomaly in this data set, and was to result from very low standard deviations amongst all of their groups as well as a small sample size in the control group. The findings of Wheeler et al. [61] were also considered an anomaly in this data set due, as well, to a small sample size. The placebo effect of injection therapies may be distinct from those not employing such interventions. The effect sizes from Wheeler et al. were not included in the calculation of mean effect size.

### Between group comparisons

The baseline pain scores in the PL group were statistically significantly greater than those in the NT group (39.18 {6.6} vs 56.4 {4.3}; t (for unequal variances) = -3.29, p = .005).

Comparisons of the change scores between the two groups (PL vs NT) at 0–10 weeks and at greater than 10 weeks for absolute (mm) or relative (%) change showed no significant differences in any of the four comparisons.

## Discussion

There are several ways to interpret the results of this analysis. The average effect size, from those studies in which this could be calculated, is in the small -to – medium range [36,37], although several studies reported virtually no change at all, especially in the placebo groups. Very few studies reported effect sizes above .80 (large effect size per Cohen [36]).

The baseline pain scores for both types of study group were in the mild-moderately severe range (3–5 out of 10). The range of average absolute changes in 100 mm pain VAS ratings is -5.7 to -11.9 mm over all outcome intervals. All but two of the studies [41,61] reported change scores of less than 20 mm. The average relative magnitudes of change ranged from -8.3% to -25.7%; however, these figures can be misleading as they do not reflect the variation in baseline scores ranging from about 30 to 80 mm.

Based on these summaries, and with due regard to the large confidence intervals for the mean effect sizes, and with due regard to the small sample in some of the sub-groups of this study, it appears that the change scores for chronic neck pain patients not receiving formal therapy increase relatively little over 4–10 weeks at which point their increase appears to plateau for up to 12 months. As well, it appears that the "placebo effect" is not significantly different from the effect obtained in unblinded no-treatment control groups.

Our findings do not appear to bear out the hypothesis that the changes obtained in the PL groups would be greater than those in NT control groups. The statistical comparisons for all four outcomes (0–10 and 11–52 weeks; mm and %) were not significant. As noted in the introduction, the basis for this hypothesis is that the "placebo effect" is postulated to be stronger than the effect obtained from mere "trial participation". This expected difference hinges on the issue of blinding: subjects assigned to no-treatment control groups become, in short order, aware of this status; this awareness is then thought to have a diminishing effect on their treatment expectations. This, along with the absence of putatively stronger "placebogenic" effects, is thought to result in the lowest expected treatment outcome.

On the other hand, subjects assigned to a placebo group are, ideally, not aware of this status; their higher treatment expectations are then thought to contribute to the more positive placebo "effect"; i.e., a short-term, self-referential state of improvement which, we had proposed, would be larger than that obtained in un-blinded, no-treatment control subjects.

The fact that our findings did not support this expected effect is, thus, surprising. This is especially so since the mean PL group baseline pain score was higher than that of the non-treatment group. One explanation may be that subjects in the no-treatment control groups, once they recognize this status, conclude that they will not, or at least may not, obtain the optimum benefit from their participation in the study. They may then decide to drop out. If they remain in the study, they may then decide to seek additional forms of relief not sanctioned by the study. These self-relief measures may include increasing the frequency of low-level over-the-counter (OTC) medications (analgesics, NSAID's, etc.), increasing the use of higher strength OTC medications, increasing the use of heat or cold applications, herbal products or other proprietary procedures, increasing the use of non-professional "treatments" such as casual massage and increasing their use of exercises, both at home and in one of the many facilities which have lately sprung up to offer rather sophisticated exercise protocols (i.e., those with guidance, high quality equipment, large variety of equipment, pleasant atmosphere, etc.).

The use of these sorts of self-treatment measures is by no means a new phenomenon in the conduct of clinical trials. Trial teams may take a variety of measures to deal with it, including, explicit attempts to limit it, attempts to measure it and attempts to incorporate it into their analytic models. It is likely that trial designers would expect that such self-help behaviors (self-directed co-interventions) would "wash-out" across all study groups. Our findings suggest that, at least for chronic neck pain subjects, this may not be so, with the difference possibly arising from the effect that un-blinded assignment to no-treatment control status has on the pain management decisions the subjects so assigned may make during their participation in the study.

As we did not intend to identify the causal explanation for the outcomes we obtained, it should be noted that, as Borghouts et al. [12] and others [3,6,9] have noted, there are many important prognostic factors in neck pain that could explain both the treated and non-treated outcomes. A specific study including some of these factors would be required to more clearly explain the lack of difference found between the two groups of studies.

Our results are very consistent with two other recent systematic reviews of placebo-controlled trials [23,24]. Hrobartsson and Gotzsche [23] reviewed 29 RCT's of a wide variety of organic and musculoskeletal pain complaints and conditions which used placebo analgesia control groups. They found that the mean effect size of these groups was 0.27 (range = -1.13 to1.07). Vase et al. [24] reviewed 23 RCT's of a wide variety of organic and musculoskeletal pain complaints and found a mean effect size of 0.15 (range = -0.95 to 0.57). Both of these studies concluded that the placebo effect is rather minimal, although Vase et al. made the point that when the placebo effect is induced more actively (rather than being used only as a control procedure), the effects are increased significantly.

Our findings are in agreement with those of Borghouts et al. [12]. The summary of the outcomes of the control groups in their review included an average level of improvement in self-rated pain scores of 14%, an average level of physician-rated improvement of 18% and an average level of self-rated "global improvement" of 40%.

Our findings are also in good accord with Farrar et al. [62] who reported that a 2-point change (30%) in the 11-point numerical pain rating scale (which is equivalent to 20 mm change in pain VAS ratings) was regarded as the minimum clinically important change by patients in a variety of pain groups. What has not, until now, been systematically determined is that at least one group of chronic pain subjects – those with neck pain in control groups, and especially those in placebo groups – does not appear to improve beyond this level.

While we attempted to obtain the largest possible sample of high quality studies, it is possible that some English-language studies were missed. As well, since non-English studies were not included, it is possible that such studies may provide different findings. However, the findings from the studies in our sample were all relatively consistent; it would be surprising to find a separate sample with greatly different findings.

The findings of our study can be of use to clinical trial planners by providing a general benchmark of minimum group mean change scores on pain measures for chronic mechanical neck pain patients. These findings may also be of use in the development of clinical guidelines, in that they provide a benchmark against which the results of studies of various treatments can be compared. This does not, however, replace using control groups in future trials, especially in the early stages of research into a specific modality of treatment. Where a body of studies does exist, and where there are no or few well-controlled trials, these findings may provide a proxy measure for guideline developers in evaluating the benefit of various treatments.

## Conclusion

Our initial question, "what is the expected outcome of chronic mechanical neck pain patients who are assigned to control groups?", has been addressed by analyzing two sets of RCT's providing data on chronic neck pain subjects in either no-treatment or placebo control groups. It appears that the changes in pain scores in subjects with chronic neck pain not due to whiplash who are enrolled in no-treatment and placebo control groups were similarly small and not significantly different. As well, they do not appear to increase over longer-term follow-up.

## Competing interests

The author(s) declare that they have no competing interests.

## Authors' contributions

HV was involved in planning and designing this study, retrieving, organizing, evaluating and selecting the article to be reviewed, abstracting the relevant data and writing all aspects of the manuscript.

KH was involved in planning and designing this study and in evaluating the articles to be included in this review. He also assisted in the writing of all aspects of the manuscript.

CH was involved in planning and designing this study and in evaluating the articles to be included in this review. She also assisted in the writing of all aspects of the manuscript.

All authors read and approved the final manuscript.

## Pre-publication history

The pre-publication history for this paper can be accessed here:



## Figures and Tables

**Table 1:** Search Strategy

1	randomized controlled trial.pt.
2	controlled clinical trial.pt.
3	randomized controlled trials.sh.
4	random allocation.sh.
5	double blind method.sh.
6	single blind method.sh.
7	1 or 2 or 3 or 4 or 5 or 6
8	clinical trial.pt.
9	exp clinical trials/
10	(clin$ adj trial$).ti, ab.
11	((singl$ or doubl$ or trebl$ or tripl$) adj (blind$ or mask$)).ti, ab.
12	placebos.sh.
13	Placebo$.ti, ab.
14	random$.ti, ab.
15	Research design.sh.
16	or/8–15
17	comparative study.sh.
18	exp evaluation studies/
19	follow up studies.sh.
20	prospective studies.sh.
21	(control$ or prospective$ or volunteer$).ti, ab.
22	or/17–21
23	7 or 16 or 22
24	neck pain.mp. or exp Neck Pain/
25	exp CERVICAL VERTEBRAE/
26	24 or 25
27	exp alternative medicine/
28	exp plants, medicinal/
29	exp plant oils/
30	exp plant extracts/
31	exp formularies, homeopathic/
32	((complementary or unconventional or folk or alternative) adj (med$ or ther$ or treat$ or care)).ti, ab.
33	exp holistic health/
34	exp Physical Therapy Techniques/
35	(physical ther$ or physiother$).ti, ab.
36	exp osteopathy/or exp osteopathic medicine/
37	(chiropract$ or naturopath$ or osteopath$ or homeopath$ or acupunct$).ti, ab.
38	or/27–37
39	23 and 26 and 38
40	limit 39 to english language

**Table 2:** No-Treatment Control Studies

**STUDY**	**CONTROL TREATMENT**	**SAMPLE SIZE OF CONTROL GROUP**	**BASELINE PAIN VAS SCORE [mean(sd)/100 mm]**	**OUTCOME INTERVAL**	ABSOLUTE CHANGE (mm on VAS)*	**RELATIVE CHANGE (%)**	**EFFECT SIZE**	**QUALITY SCORE (Jadad Scale out of 5)**
Chiu et al., 2004 [58]	Infrared therapy and advice	78	43 (21)	6 weeks 6 months	-5 -4	-11.7% -10.1%	.22 .17	3.5
Waling et al., 2000 [40]	Education sessions for 2 hr, 1/week for 10 weeks	21	32 (23)	10 weeks	-2	-6.2	.08	3
Viljanen et al., 2003 [43]	No treatment	130	41 (22)	12 weeks 24 weeks 52 weeks	-14 -12 -9	-34.1 -29.2 -21.9	.59 .48 .38	3
Taimela et al., 2000 [41]	No formal treatment	26	59.9 (19.6)	12 weeks 52 weeks	-20.9 not given	-33% undet.	undet undet	3
Lundblad et al., 1999 [58]	No treatment	23	Usual pain = 20 (14) Worst pain = 55 (28)	6 weeks usual: worst:	-9 -7	-45 -13	.64 .25	3
TOTAL/AVERAGE		278	46.2		For 1–10 wk = -5.7 [-8.6, -2.8] mm For 12–52 wk = -11.9 [-17.4, -6.4] mm	For 1–10 wk = -10.3% [-14.4, -6.2]] For 12–52 wk = -25.7% [-34.3, -17.1]	For 1–10 wk = .18 [-0.05, 0.41] For 12–52 wk = .4 [0.12, 0.68]	

Caption: sd = standard deviation; undet = undetermined

**Table 3:** Placebo-control studies

**STUDY**	**CONTROL TREATMENT**	**SAMPLE SIZE OF CONTROL GROUP**	**BASELINE PAIN VAS SCORE [mean(sd) or median/100 mm]**	**OUTCOME INTERVAL**	ABSOLUTE CHANGE (mm on VAS)*	**RELATIVE CHANGE (%)**	**EFFECT SIZE**	**QUALITY SCORE (Jadad Scale out of 5)**
Trock et al., 1994 [49]	Placebo EMT, 3–5/week for four weeks	29	62.3 (24.16)	2 weeks 4 weeks 8 weeks	-12 -16.3 -14.7	-19 -26 -24	.44 .67 .54	5
Sator-Katzensch-lager et al., 2003 [50]	Control auricular acupuncture, 1 Tx per week for 6 weeks	11	71 (3)	6 weeks 10 weeks	-16 -4	-22 -5	5.3* .99	5
Gur et al., 2004 [59]	Placebo laser, 10 treatments in 2 weeks	26	72 (2.52)	2 weeks 3 weeks 12 weeks	-5.7 -11.7 +.04	-8 -16 +1	.14 .26 +.01	4.5
Rigato et al., 2002 [47]	Placebo electrotherapy daily for 15 days	50	High = 80 Med = 48 Low = 36 (no sd's)	7,15 & 45 days	**At 7 days** High = 0 Med = 0 Low = -8 **At 15 days** High = 0 Med = 0 Low = 0 **At 45 days** High = 0 Med = +2 Low = +2	**At 7 days** High = 0 Med = 0 Low = -22 **At 15 days** High = 0 Med = 0 Low = 0 **At 45 days** High = 0 Med = +4 Low = +6		4
Snyder-Mackler et al., 1989 [52]	Placebo laser, 3 Tx over 9 days	11	55 (no sd)	9 days	-3	-5		4
Foley-Nolan et al., 1990 [29]	Placebo EMT for 3 weeks	10	67.5 (median)	3 weeks	-12.5	-19		4
Hong et al., 1982 [60]	Non-magnetic necklace for 3 weeks	25	71 (14) [converted from 4-point scale]	3 weeks	-7	-10	.05	4
Thorsen et al., 1992 [30]	Placebo laser, 6 Tx over 2 weeks; then, cross-over	47	21 (median)	3 weeks	-10	-48		3.5
Wheeler et al., 1998 [61]	1–2 botox injections	11	65 (5) [NPDS/100*]	3 weeks 16 weeks	-16 -21	-24.6 -32.2	2.2* 2.6*	3
Ceccherelli et al., 1989 [28]	Placebo laser for 12 Tx over 24 days	14	29.2 (12.6)	4 weeks 12 weeks	+8.2 +6.4	+28 +22	-.56 -.40	3
Nabeta and Kawakita, 2002 [54]	Sham acupuncture, 3 Tx over 3 weeks	17	48.8 (27.9)	4 weeks	-2	-4	.07	3
Irnich et al., 2001 [55]	Sham laser acupuncture, 5 Tx over 3 weeks	57	57.15 (26.71)	4 weeks	-17.28 [10–24.6]	-30		3
He et al., 2004 [51]	Sham acupuncture, 10 Tx over 4 weeks	12	48 (9)	4 weeks 24 weeks	-12.1 -12.1	-24 -24	1.4 1.4	3
Ozdemir et al., 2001 [53]	Placebo laser, daily for 10 days	30	73 (14)	10 days	-5	-7	.04	3
TOTAL/AVERAGE		350	56.6		For 0–10 weeks = -5.0 [-8.1, -1.9] mm For 12–24 weeks = -6.7 [-18.6, 5.2] mm	For 0–10 weeks = -10.6% [-16.4, -4.8] For 12–24 weeks = -8.3% [-32.4, 15.8]	For 1–10 weeks = .50 [0.10, 0.9] For 12–24 weeks = .33 [-1.97, 2.6]	

Caption:

wk = week; Tx = treatment; EMT = electromagnetic therapy; TENS = transcutaneous electrical nerve stimulation; * = not included in analysis
